# Multi-Walled Carbon Nanotube Array Modified Electrode with 3D Sensing Interface as Electrochemical DNA Biosensor for Multidrug-Resistant Gene Detection

**DOI:** 10.3390/bios13080764

**Published:** 2023-07-27

**Authors:** Ruiting Chen, Hejing Chen, Huaping Peng, Yanjie Zheng, Zhen Lin, Xinhua Lin

**Affiliations:** Higher Educational Key Laboratory for Nano Biomedical Technology of Fujian Province, Department of Pharma-Ceutical Analysis, Faculty of Pharmacy, Fujian Medical University, Fuzhou 350122, China; ruiting_chen@fjmu.edu.cn (R.C.); jane_0606@fjmu.edu.cn (H.C.); gillzheng@fjmu.edu.cn (Y.Z.); zhenlin12@fjmu.edu.cn (Z.L.)

**Keywords:** multidrug resistant gene, multi-walled carbon nanotube array, electrochemical DNA biosensor, 3D sensing interface

## Abstract

Drug resistance in cancer is associated with overexpression of the multidrug resistance (MDR1) gene, leading to the failure of cancer chemotherapy treatment. Therefore, the establishment of an effective method for the detection of the MDR1 gene is extremely crucial in cancer clinical therapy. Here, we report a novel DNA biosensor based on an aligned multi-walled carbon nanotube (MWCNT) array modified electrode with 3D nanostructure for the determination of the MDR1 gene. The microstructure of the modified electrode was observed by an atomic force microscope (AFM), which demonstrated that the electrode interface was arranged in orderly needle-shaped protrusion arrays. The electrochemical properties of the biosensor were characterized by cyclic voltammetry (CV), differential pulse voltammetry (DPV), and electrochemical impedance spectroscopy (EIS). Chronocoulometry (CC) was used for the quantitative detection of the MDR1 gene. Taking advantage of the good conductivity and large electrode area of the MWCNT arrays, this electrochemical DNA sensor achieved a dynamic range from 1.0 × 10^−12^ M to 1.0 × 10^−8^ M with a minimal detection limit of 6.4 × 10^−13^ M. In addition, this proposed DNA biosensor exhibited high sensitivity, selectivity, and stability, which may be useful for the trace analysis of the MDR1 gene in complex samples.

## 1. Introduction

Cancer is one of the leading causes of death, and has become a serious threat to human health worldwide in recent years. Multidrug resistance (MDR) is the main factor leading to the failure of chemotherapy, which has been one of the most effective cancer treatments [1]. The over-expression of the human MDR1 gene, which encodes a plasma membrane multidrug efflux transporter P-glycoprotein (P-gp), was believed to be the main reason for MDR [2]. P-gp uses the energy released from ATP hydrolysis to efflux drugs across cell membranes, leading to a decrease in the intracellular concentration of anticancer drugs [3]. The expression of MDR1 is mainly determined by regulatory changes in the transcription or stability of MDR1 mRNA [4]; therefore, the detection of mRNA is particularly important for the study of the level of MDR of tumor cells, thus providing important guidance for cancer chemotherapy in clinical treatment [5]. Taking into account the instability of mRNA, the corresponding cDNA obtained by reverse transcription was used as the detection target for the purposes of our study.

Various techniques have been adopted to detect MDR, including reverse transcriptase polymerase chain reaction (RT-PCR) [6], western blot [7], northern blot [8], slot blot [9], immunocytochemistry [10], and flow cytometry [11]. The classical methods have many limitations and shortages, such as being expensive, complicated to operate, and time-consuming, as well as having low sensitivity. A more convenient method was demanded for MDR detection. The electrochemical DNA biosensor has received considerable attention due to its excellent performance, which is suitable for the rapid, sensitive, and specific detection of DNA [12,13,14,15,16,17]. Currently, various electrochemical DNA biosensors have been reported for the analysis of the human MDR1 gene [18,19,20,21].

Nanomaterials have been widely used in biological sensing research due to their extraordinary properties. Electrochemical biosensors modified with nanomaterials demonstrate greatly improved performance. Carbon nanotubes, especially, as a traditional nanomaterial, have a high specific surface area and good conductivity and biocompatibility, all of which make them most suitable for modification with biosensors [22,23,24,25,26,27]. Among carbon nanotubes, multi-walled carbon nanotubes (MWCNTs) are cheap and generally available industrial products that can reduce the cost of a biosensor.

**Figure 1:**
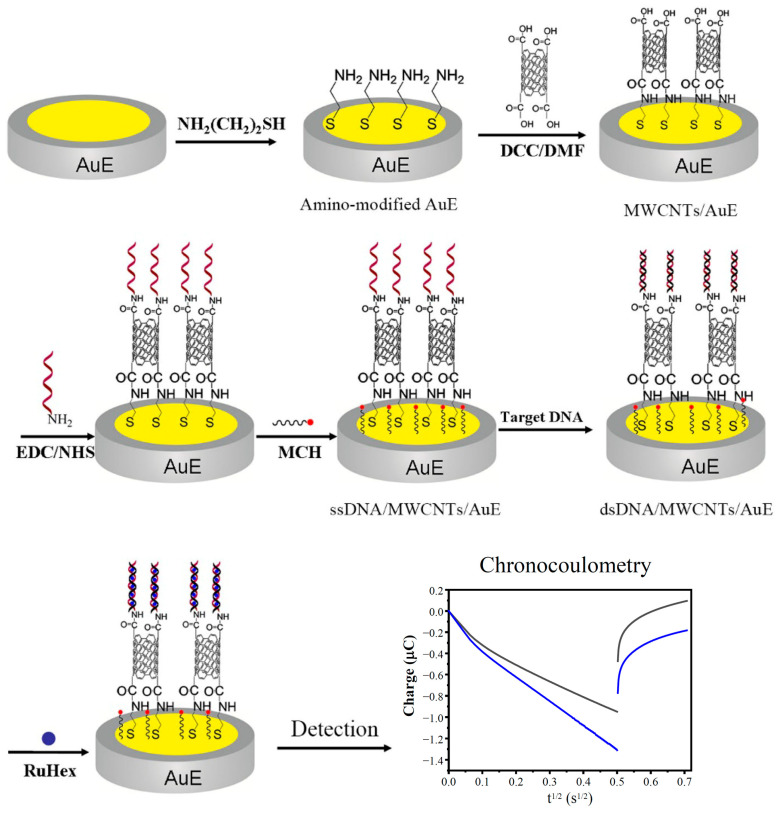
Schematic illustration of the fabrication of the electrochemical DNA biosensor.

In this paper, an electrochemical DNA biosensor based on the modification of MWCNT arrays has been designed and constructed, one depending on electrochemical sensing technology and nano techniques for the effective detection of the human MDR1 gene. The approach to the construction and detection of the biosensor is shown in Figure 1. The amino groups were linked to the gold electrode (AuE) by means of a reaction with the cysteamine via Au-S bond, and then connected with the carboxylic MWCNTs by amide linkage to form an aligned MWCNT-array modified electrode. The 5’ end-labeled capture-probe ssDNA with amino group was immobilized at the end of the carboxylic MWCNT pipe by amide bond formation, and then the surface of bare gold was blocked with 6-Mercapto-1- Hexanol (MCH) to construct the DNA biosensor. The sequence of probe ssDNA is set as a complementary strand of cDNA in order to achieve the highly selective detection of cDNA. In the further study of the real sample assay, the total RNAs were extracted after genome elimination and then reversed to cDNA using a commercial kit, after which the reversed cDNA was detected using our proposed method. In this way, the MDR1 mRNA could be indirectly detected with robustness.

The MWCNT array’s modified electrodes have certain advantages, as follows. First, MWCNTs vertical to the substrate were orderly arranged to form a three-dimensional array, which can greatly extend the surface area of the electrode and then increase the loading of probe ssDNA to improve the sensitivity of the biosensor. Second, MWCNTs with high electronic conductivity formed electron transmission channels connecting the probe ssDNA and the gold electrode, which can accelerate the electronic transfer of the biosensor. Third, the biocompatibility of MWCNTs was beneficial in maintaining the biological activities of the probe ssDNA. Fourth, a covalent bond between the probe ssDNA and the gold electrode constructed a more stable sensing interface, which can improve the stability and reproducibility of the biosensor.

## 2. Materials and Methods

### 2.1. Chemicals and Reagents

Multi-walled carbon nanotubes (MWCNTs, diameter: 10–20 nm, length: 5–15 μm), cysteamine, dicyclohexylcarbodiimide (DCC), 1-ethyl-3- (3-dimethyl aminopropyl) carbodiimide (EDC), and N-Hydroxysuccinimide (NHS) were purchased from Aladdin (Shanghai, China). Hexaammineruthenium chloride (RuHex) and 6-Mercapto-1- Hexanol (MCH) were purchased from Sigma (St. Louis, MO, USA). H_3_PO_4_, K_3_[Fe(CN)_6_] and K_4_[Fe(CN)_6_]·2H_2_O were purchased from Sinopharm Chemical Reagent Co. Ltd. (Shanghai, China).

All oligonucleotides used in this work were synthesized and purified by TaKaRa Inc. (Dalian, China), and the sequences are shown in Table 1.

The buffer solutions used in this study were as follows: the buffer for immobilization of the probe ssDNA contained 10 mM Tris-HCl, 1 mM ethylenediaminetetraacetic acid (EDTA), and 1 M NaCl (pH 8.0). The hybridization buffer was a 10 mM phosphate buffer solution (PBS) containing 1 M NaCl (pH 7.4).

### 2.2. Preparation of Carboxylic MWCNTs

Commercial MWCNTs are about 5–15 μm long and have chemical inertia. Therefore, they should be cut off in a strong acid solution and modified by a carboxyl group at the open end before use in electrode modification. The acidization process is as follows [28]. The mixed-acid solution of H_2_SO_4_ (98%) and HNO_3_ (70%) was prepared in a volume ratio of 3:1 and then commercial MWCNTs were added to the mixed acid. Ultrasonic dispersal of the suspension for 30 min at room temperature then ensued, followed by raising the temperature to 60 °C; it was then kept at this temperature for 4 h under ultrasonic vibration. The product was poured into double-steamed water and filtered with a 0.22 mm microporous membrane, and then repeatedly washed with double-steamed water until pH was neutral, and subsequently vacuum-dried 24 h at 60 °C. SEM images (Figure S1 in Supplementary Materials) and AFM images (Figure S2 in Supplementary Materials) of commercial MWCNTs and acid-treated MWCNTs shows that the MWCNTs were successfully cut off. The stretching vibration band of carbonyl in the IR spectrum indicates that the Carboxylation of the MWCNTs (Figure S3 in Supplementary Materials).

### 2.3. Preparation of Amino-Modified AuE

Amino-modified AuE was prepared by reaction with cysteamine via the Au-S bond [29,30]. AuE was successively ultrasonicated in a piranha solution (H_2_SO_4_/H_2_O_2_: 3/1) and doubly distilled water at room temperature for 10 min before being wet polished to mirror-like surface with alumina slurry of 0.3 and 0.05 mm. The electrode was then successively ultrasonically cleaned by 1:1 nitric acid, acetone, and doubly distilled water, respectively. The pre-processed AuE was immersed in an ethanol solution containing 1 mM cysteamine at room temperature for 24 h, then cleaned with doubly distilled water and dried with nitrogen.

### 2.4. Preparation of MWCNTs/AuE

MWCNTs/AuE were prepared by reaction of carboxylic MWCNTs and amino-modified AuE in the presence of DCC as the coupling agent [29,30]. A quantum of 10 mg carboxylic MWCNTs and 20 mg DCC were added to 10 mL of N, N- dimethylformamide (DMF), and then subjected to ultrasonic dispersion at room temperature for 10 min; MWCNTs/AuE were obtained by soaking amino-modified AuE in the mentioned solution at 60 °C for 9 h, and then cleaning with doubly distilled water and dried with nitrogen. The effects of reaction time on the modification of MWCNTs were as shown in Figure S4 in Supplementary Materials.

### 2.5. Preparation of ssDNA/MWCNTs/AuE

MWCNTs/AuE were immersed in a mixture solution of EDC and NHS at room temperature for 30 min to activate the carboxyl groups that were located at the end of the carboxylic MWCNTs pipe, washed with doubly distilled water, and then dried with nitrogen to reserve. Dropwise, 4 µL 5 µM amino-modified probe ssDNA-1 (P1) was added to the surface of the AuE at room temperature for 1.5 h to form an amide bond with carboxyl groups of MWCNTs. The probe ssDNA was covalently linked onto the pipe end of the MWCNTs. The gold electrode modified with ssDNA was immersed in blocking buffer containing 200 µL MCH at room temperature for 1 h to block the bare gold surface, and then washed with doubly distilled water and dried with nitrogen. Finally, we obtained a DNA biosensor marked as ssDNA/MWCNTs/AuE. Effects of the reaction time and probe concentration of the probe ssDNA immobilization were as shown in Figure S5 in Supplementary Materials.

### 2.6. Hybridization of the DNA Biosensor

The hybridization procedure was performed by immersing the ssDNA/MWCNTs/AuE in 10 mM PBS containing target ssDNA at 40 °C for 1 h to form double-stranded DNA (dsDNA). The hybridized electrode was rinsed with doubly distilled water to remove the nonspecifically adsorbed ssDNA. The electrode obtained was marked as dsDNA/MWCNTs/AuE. Effects of hybridization temperature and hybridization time were as shown in Figure S6 in Supplementary Materials.

### 2.7. Instrument and Apparatus

A traditional three-electrode system had been set up for the electrochemical characterization in which the modified electrode, the Ag/AgCl electrode, and platinum wire electrode were used as the working electrode, reference electrode, and auxiliary electrode, respectively. Electrochemical measurements for cyclic voltammogram (CV), electrochemical impedance spectroscopy (EIS), differential pulse voltammetry (DPV), and chronocoulometry (CC) were performed on an Autolab PGSTAT302F electrochemical workstation (Metrohm, The Netherlands).

### 2.8. Quantitative Analysis of Chronocoulometry

The modified electrode was immersed in a pH 7.4 Tris-HCl buffer containing 50 μM [Ru(NH_3_)_6_]Cl_3_ for 30 min and then the charge on the electrode surface was measured by chronocoulometry. Perform CC readouts indicated the following parameters: initial potential: 0.2 V; final potential: −0.5 V; pulse width: 0.25 s; sample interval: 0.002.

## 3. Results and Discussion

### 3.1. Morphology Characterization of MWCNTs/AuE

In order to observe the surface morphology of the MWCNTs array modified electrode more intuitively, we used a detachable gold electrode in the experiment. MWCNTs/AuE was prepared according to the method mentioned above in Section 2. After the electrode was modified, the gold sheet could be disassembled and used directly for AFM characterization.

**Figure 2:**
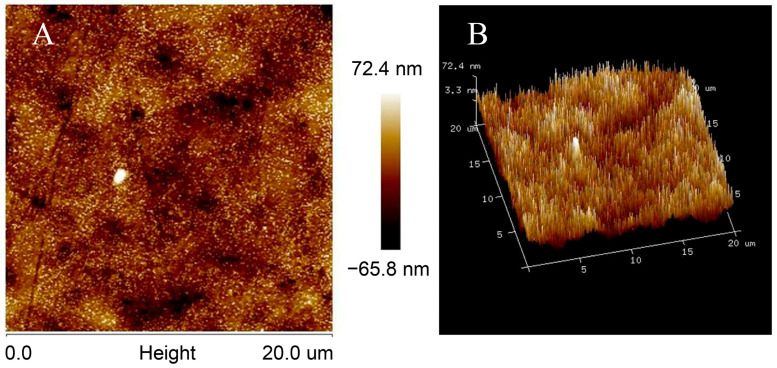
AFM images of MWCNTs/AuE, including 2D images (**A**) and 3D images (**B**).

The morphology of the surface of MWCNTs/AuE was as shown in Figure 2; the ordered needle-shaped protrusion arrays on the surface of the MWCNTs/AuE indicated that the carboxyl group of MWCNTs located in the end of the tube pipe had been successfully connected to the amino-modified AuE. The MWCNTs remain upright on the gold surface, and an ordered three-dimensional array nanostructure is constructed, greatly extending their specific area and increasing the loading of probe ssDNA, which will improve the sensitivity of the biosensor. The covalent bonds between MWCNTs and gold surfaces are much stronger than those of the molecular forces, and the electrostatic force contributed to the building of a more stable sensing interface, which improved the stability and reproducibility of the biosensor. The MWCNT arrays on the gold’s surface formed a conducting layer with a thickness of about 50 nm, according to the AFM images. MWCNTs are conductor wires that connect probe ssDNA and the gold’s surface directly to accelerate the electron transfer of the electrode surface. The space between MWCNTs can be an accelerated mass transfer process, which contributes to the rapid detection of the biosensor.

### 3.2. Electrochemical Characterization

Cyclic voltammogram (CV), differential pulse voltammetry (DPV), and electrochemical impedance spectroscopy (EIS) were employed to explore the electrochemical features of the modified electrode’s surface. Electrochemical characterization was performed in an aqueous solution containing 0.1 M KCl and 10 mM K_3_[Fe(CN)_6_]/K_4_[Fe(CN)_6_].

**Figure 3:**
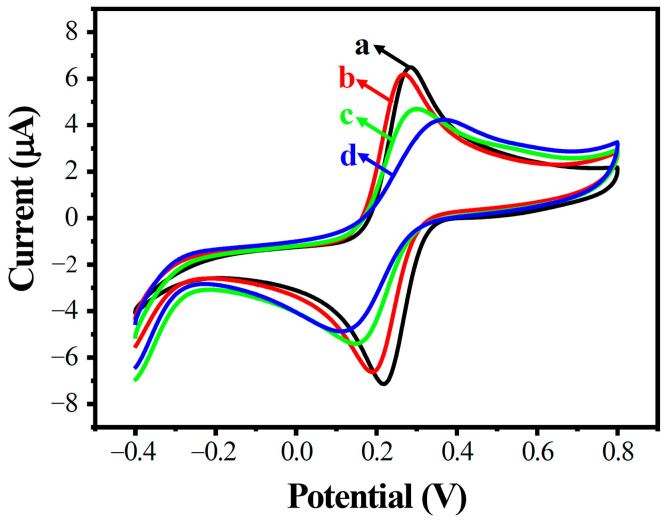
Cyclic voltammogram diagram of modified electrodes in different states. AuE (a, black), MWCNTs/AuE (b, red), ssDNA/MWCNTs/AuE (c, green), and dsDNA/MWCNTs/AuE (d, blue).

CVs in the potential range of −0.4 to 0.8 V are shown in Figure 3. Two well-defined redox peaks were observed at AuE and MWCNTs/AuE, respectively (curves a and b); the peak potential differences increased and the peak electric current decreased accordingly when probe ssDNA was immobilized (ssDNA/MWCNTs/AuE, curve c), and the peak electric current decreased further after hybridization with target ssDNA (dsDNA/MWCNTs/AuE, curve d). This result indicated that the negatively charged DNA phosphate skeleton is blocked by the electronegative [Fe(CN)_6_]^3−/4−^, forming an electron-transfer and mass-transfer blocking layer to inhibit the reaction on the electrodes.

**Figure 4:**
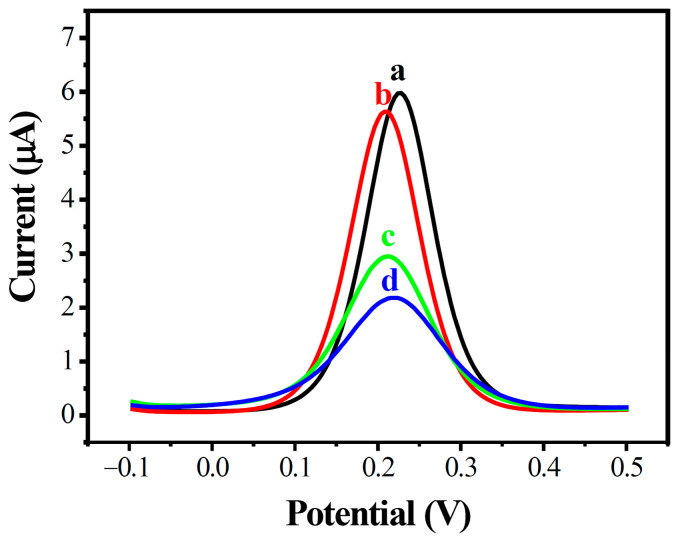
Differential pulse voltammetry diagram of modified electrodes in different states. AuE (a, black), MWCNTs/AuE (b, red), ssDNA/MWCNTs/AuE (c, green), and dsDNA/MWCNTs/AuE (d, blue).

The construction processes of the modified MWCNT array electrode were further confirmed by DPV analysis, as shown in Figure 4. A similar phenomenon was observed in DPVs in the potential range of −0.1 to 0.5 V; the peak electric current decreased greatly with increased amounts of DNA on the electrode’s surface.

EIS was used to track the conductivity of different sensing interfaces for stepwise modification processes. A typical impedance spectrum plot was created, including two parts presented in the form of the Nyquist plot. The semicircle at higher frequencies corresponds to the electron transfer process, and the linear tail at lower frequencies corresponds to the diffusion process. The electron-transfer resistance R_et_ is represented by the diameter of the semicircle, which is the most sensitive parameter with respect to changes in the electrode’s interface.

**Figure 5:**
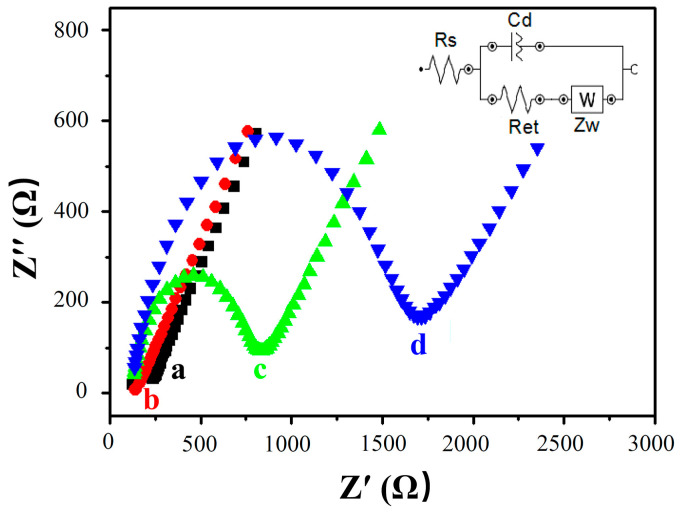
Electrochemical impedance spectroscopy of modified electrodes in different states. AuE (a, black), MWCNTs/AuE (b, red), ssDNA/MWCNTs/AuE (c, green), and dsDNA/MWCNTs/AuE (d, blue).

Figure 5 shows the impedance spectra of different modified electrodes. A Small R_et_ was observed with the AuE (curve a), and the R_et_ was further reduced when the MWCNTs were assembled on the AuE, because of the good conductivity of MWCNTs and the increment of the specific surface area, which accelerated the electron transfer of the electrode’s surface (MWCNTs/AuE, curve b). After immobilization of the probe ssDNA, R_et_ suddenly increased to 828 Ω (curve c) due to the rejection of DNA phosphate skeleton being negatively charged with electronegative [Fe(CN)_6_]^3−/4−^, impeding the transmission of electrons on the electrode’s surface. When the target ssDNA was hybridized with the probe ssDNA to form dsDNA, R_et_ increased further to 1694 Ω due to more electronegative DNA being linked to the electrode surface (curve d). These results indicated that the DNA biosensor had been successfully built and could be hybridized with the target ssDNA as expected.

### 3.3. RuHex and Quantitative Analysis of Chronocoulometry

**Figure 6:**
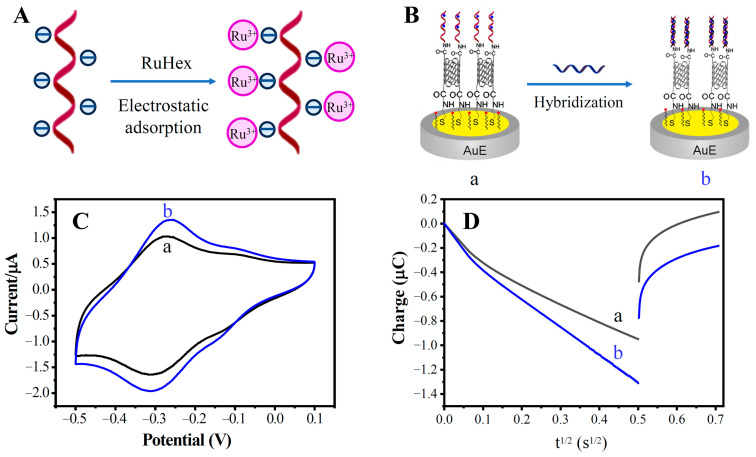
Electrochemical behavior and characterization of the RuHex indicator. (**A**) RuHex electrostatic adsorption diagram; (**B**) schematic diagram of DNA hybridization; (**C**) cyclic voltammogram diagram of ssDNA/MWCNTs/AuE (a) and dsDNA/MWCNTs/AuE (b); (**D**) CC curves of ssDNA/MWCNTs/AuE (a) and dsDNA/MWCNTs/AuE (b).

As shown in Figure 6A, the phosphate backbone of DNA is negatively charged. RuHex with a positive charge can be combined with the DNA phosphate skeleton by electrostatic force, which can be used as an electrochemical hybridization indicator [31,32]. Chronocoulometric interrogation of the redox reaction of RuHex quantitatively reflects the number of DNA strands located at the electrode’s surface. Figure 6B shows the schematic diagram of the probe ssDNA capturing target ssDNA. With the capture of the target ssDNA, the number of DNA strands localized at the electrode surface increased, and the amount of adsorbed RuHex also increased. After DNA hybridization, a stronger peak current signal can be seen on the CV curve (Figure 6C). The chronocoulometry (CC) can produce stronger signals than can the CV method in the system with RuHex as hybridization indicator [31]. As shown in Figure 6D, the CC curves showed significant differences before and after hybridization, indicating an increase in the electrode’s surface charge. The calculation of the electric quantity Q refers to the literature. [33,34]. The charge increment ΔQ is related to the amount of target ssDNA captured, which is associated with the concentration of the target ssDNA in solution. Therefore, CC was employed for the quantitative analysis of the target ssDNA.

### 3.4. Signal Amplification of MWCNTs Modification

ssDNA/AuE and ssDNA/MWCNTs/AuE were used to investigate the signal amplification of MWCNTs modification. ssDNA/AuE was prepared by dropping the mercapto-modified probe ssDNA-2 (P2) onto AuE, which can be linked to the gold electrode surface by the formation of Au-S bonds. ssDNA/MWCNTs/AuE was prepared according to Figure 1. The hybridization procedure was carried out by immersing the electrodes in 10 mM PBS containing 1nM complementary ssDNA at 40 °C for 1 h.

Electric quantity increments (ΔQ) of DNA hybridization related to the loading of the probe ssDNA and to the concentration of the target ssDNA. Under an equal concentration of target ssDNA, the higher ΔQ means higher loading of the probe ssDNA.

**Figure 7:**
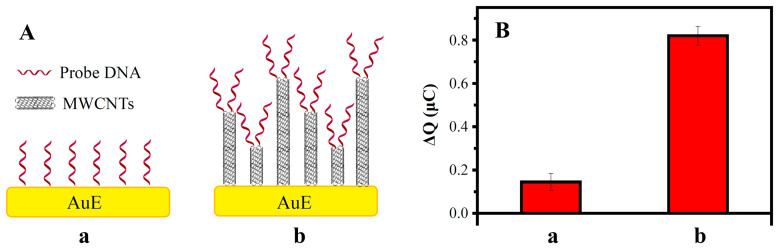
Effect of the modification of the electrode surface in the charge increment ΔQ: (**A**) schematic diagram of ssDNA/AuE (a) and ssDNA/MWCNTs/AuE (b); (**B**) ΔQ of ssDNA/AuE (a) and ssDNA/MWCNTs/AuE (b).

Figure 7A shows a schematic diagram of the probe ssDNA loaded on the electrode’s surface. When the probe ssDNA is directly bonded to the surface of the gold electrode, the loading capacity is limited by the steric hindrance of DNA and the specific surface area of the electrode. When MWCNTs are bonded on the surface of the gold electrode, MWCNTs of different lengths form uneven 3D sensing interfaces, which greatly increases the specific surface area and eases the steric hindrance of DNA, thus increasing the load of the probe ssDNA.

The ΔQs of the ssDNA/AuE and ssDNA/MWCNTs/AuE are shown in Figure 7B. The ΔQ increased to five times after the modification of the MWCNTs due to the high loading of the ssDNA probes caused by the increase in the specific surface area. The amplification of the electrochemical signal can greatly improve electrical responses and the detection sensitivity of the biosensor, which was beneficial for the detection of trace amounts of the MDR1 gene.

### 3.5. Specificity and Reproducibility of DNA Biosensor

**Figure 8:**
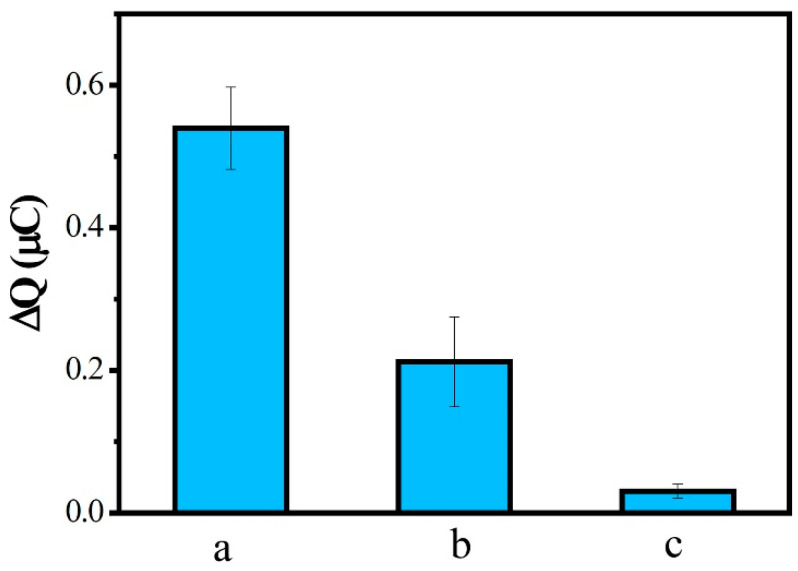
ΔQ of ssDNA/MWCNTs/AuE after hybridization with complementary sequence (a), one-base mismatch sequence (b), and noncomplementary sequence (c).

Specificity is a key factor in the evaluation of the performance of a biosensor. Biosensor specificity experiments were performed using a concentration of 0.1 nM complementary sequence (a), one-base mismatch sequence (b), and non-complementary sequence (c) separately hybridized with probe ssDNA, and then measured by CC. As shown in Figure 8, ΔQ after hybridization of the one-base mismatch sequence and the non-complementary sequence corresponded to 39.25% and 5.61% of the complementary sequence, respectively. These results indicate that the DNA biosensor we built had a good specificity in distinguishing the MDR1 gene from even a single-base mismatched sequence.

To evaluate the reproducibility of the DNA biosensor, a 1 nM complementary target solution was used in parallel experiments five times; the relative standard deviation of measurements is 5.3%, showing that the fabricated DNA biosensor performed with high reproducibility.

### 3.6. Quantitative Analytical Performance

**Figure 9:**
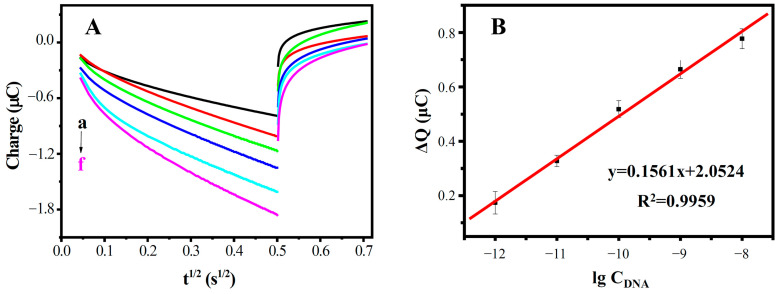
(**A**) CC curves of different concentrations of complementary ssDNA (from a to f: 0, 10^−12^, 10^−11^, 10^−10^, 10^−9^ and 10^−8^ M); and (**B**) the standard curve of the biosensor.

Under optimal conditions, the quantitative analysis of the DNA biosensor was investigated using the probe ssDNA to hybridize with the different concentrations of the target ssDNA sequences. Figure 9 shows the CC curves of DNA/MWCNTs/AuE at various concentrations of complementary target ssDNA. As expected, the electric quantity increased with the concentration of the complementary target ssDNA. The ΔQ were linear with the logarithm of the complementary target ssDNA concentrations in the range from 1.0 × 10^−12^ M to 1.0 × 10^−8^ M, with a detection limit of 6.4 × 10^−13^ M. The linear equation is ΔQ = 0.1561lgC_DNA_ + 2.0524. The result shows that the proposed DNA sensor has good analytical performance in the detection of the MDR1 gene.

The linear range and detection limit of this DNA biosensor was compared with those published in previous similar work (Table 2), showing that the proposed DNA biosensor has good analytical performance for specific sequences of DNA detection.

## 4. Conclusions

This work has designed and constructed a novel three-dimensional electrochemical DNA biosensor for rapid detection of the MDR1 gene based on a modified MWCNT array electrode. The surface morphology of the modified electrode has been investigated by AFM. CV, DPV, and EIS were employed to explore the electrochemical features of the electrode. CC was used for quantitative analysis of the MDR1 gene. Under optimal conditions, the increases in electric quantity were linearly related to the logarithm of the target ssDNA concentrations from 1.0 × 10^−12^ M to 1.0 × 10^−8^ M, with a detection limit of 6.4 × 10^−13^ M (S/N of 3). The biosensor has high specificity, sensitivity, and stability. To further evaluate the application potential of the DNA biosensor, future research should focus on applications in clinical diagnosis.

## Figures and Tables

**Table 1:** Synthetic oligonucleotide sequences.

Synthetic Oligonucleotide	DNA Sequences
Probe ssDNA-1 (P1)	5’-NH_2_-(CH_2_)_6_-TTC CTT CTT ATC TTT TTC ACT TTT ATT GTT-3’
Probe ssDNA-2 (P2)	5’-SH-(CH_2_)_6_-TTC CTT CTT ATC TTT TTC ACT TTT ATT GTT-3’
Complementary ssDNA	5’-AAC AAT AAA AGT GAA AAA GAT AAG AAG GAA-3’
One-base mismatch ssDNA	5’-AAC AAT AAA AGT GAA AGA GAT AAG AAG GAA-3’
Non-complementary ssDNA	5’-CGA CCG TGC CTC AGC CTG CTA TCA CTA CCG-3’

**Table 2:** Comparison of the linear ranges and detection limits of the different electrochemical DNA sensors.

Modified Electrodes	Detection Method	Linear Range (M)	LOD (M)	Ref.
DNA-Tb(QS)_3_	DPV	3.0 × 10^−8^–1.85 × 10^−7^	2.1 × 10^−8^	[12]
DNA/GO/CoFe_2_O_4_/ZnAl-LDH/FTO	DPV	2.0 × 10^−7^–1.0 × 10^−5^	1.0 × 10^−9^	[13]
Ni-MOF composite/AuNPs/CNTs	DPV	1.0 × 10^−8^–1.0 × 10^−6^	1.3 × 10^−10^	[14]
lambda exonuclease	EIS	1.0 × 10^−10^–2.0 × 10^−8^	4.2 × 10^−11^	[15]
Au NPs/TB–GO/GCE	DPV	1.0 × 10^−11^–1.0 × 10^−9^	2.9 × 10^−12^	[19]
DNA/MWCNTs/Cys/AuE	CC	1.0 × 10^−12^–1.0 × 10^−8^	6.4 × 10^−13^	This work

## Data Availability

Not applicable.

## Supplementary Materials

The following supporting information can be downloaded at: https://www.mdpi.com/article/10.3390/bios13080764/s1, Figure S1: SEM images of commercial MWCNTs (A) and acid-treated MWCNTs (B); Figure S2: AFM images of commercial MWCNTs and acid-treated MWCNTs; Figure S3: IR spectra of commercial MWCNTs (a) and acid-treated MWCNTs (b); Figure S4: The effects of reaction time on the modification of MWCNTs; Figure S5: The effects of the reaction time (A) and probe concentration (B) of the probe DNA immobilization on the response of the biosensor; Figure S6: The effects of hybridization temperature (A) and hybridization time (B) on the response of the biosensor.
