# Supplementary material for: Multi-Walled Carbon Nanotube Array Modified Electrode with 3D Sensing Interface as Electrochemical DNA Biosensor for Multidrug-Resistant Gene Detection
Source: Biosensors (Basel). 2023 Jul 27;13(8):764. doi: 10.3390/bios13080764 (PMC10452495; doi:10.3390/bios13080764)
Supplement: Supplementary file 1 [file biosensors-13-00764-s001.zip › biosensors-2455021-supplementary.pdf]

Supporting Information

Multi-Walled Carbon Nanotube Array Modified Electrode with 3D Sensing Interface as Electrochemical DNA Biosensor for Multidrug-Resistant Gene Detection

Ruiting Chen †, Hejing Chen †, Huaping Peng *, Yanjie Zheng, Zhen Lin and Xinhua Lin *

Higher Educational Key Laboratory for Nano Biomedical Technology of Fujian Province, Department of Pharma-Ceutical Analysis, Faculty of Pharmacy, Fujian Medical University, Fuzhou 350122, China; ruiting_chen@fjmu.edu.cn (R.C.); jane_0606@fjmu.edu.cn (H.C.); gillzheng@fjmu.edu.cn (Y.Z.); zhenlin12@fjmu.edu.cn (Z.L.)

* Correspondence: penghuaping@fjmu.edu.cn (H.P.); xhl1963@fjmu.edu.cn (X.L.)

† These authors contributed equally to this work.

1. Characterization of carboxylic MWCNTs

1.1 SEM images

Morphology characterizations of MWCNTs before and after the acidizing process were characterized by SEM. SEM images of commercial MWCNTs (A) and acid-treated MWCNTs (B) are shown in **Figure S1**. The tubes of commercial MWCNTs are obviously longer than those of acid-treated ones. Many open ends of acid-treated MWCNTs were observed in the SEM image of acid-treated MWCNTs, indicating that commercial MWCNTs have been successfully cut off into small fragments under the acidification treatment.

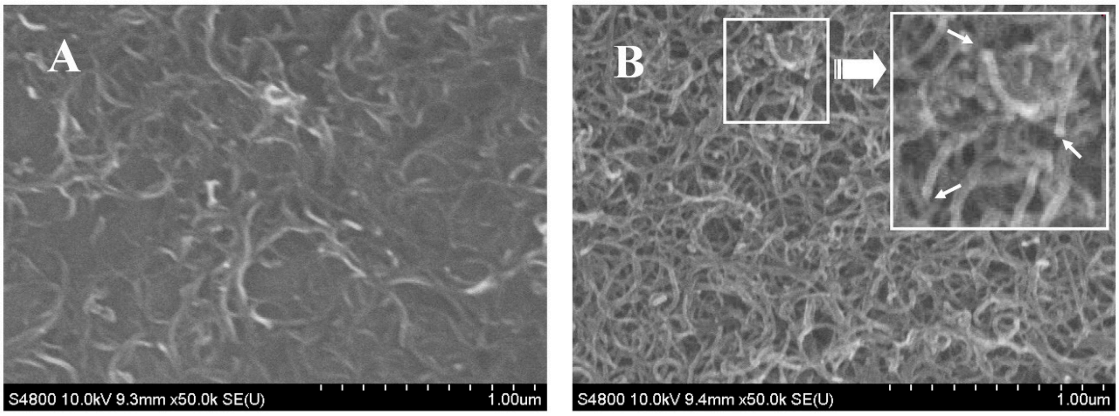

Figure S1. SEM images of commercial MWCNTs (A) and acid-treated MWCNTs (B). Upper right: amplification images of the acid-treated MWCNTs' open ends.

1.2 AFM images

Morphology characterization of MWCNTs before and after the acidizing process were characterized by AFM. Commercial MWCNTs and acid-treated MWCNTs were ultrasonically dispersed in dimethylformamide to prepare the sample suspension, and the samples were dropped onto freshly peeled mica sheets and dried naturally for AFM characterization.

The AFM images of commercial MWCNTs (A and B) and acid-treated MWCNTs (C and D) are shown in **Figure S2**. Commercial MWCNTs have a diameter of 10-20 nm and a length

of 5-15 μm. After being acid treated, they were cut to a length of 30-500 nm. As free MWCNTs, commercial MWCNTs, and acid-treated MWCNTs both lie flat on the mica substrate; they differ from the bonded MWCNTs, which remain upright on the gold's surface. Experiments show that short MWCNTs bond more easily with the gold surface, thus forming orderly needle-like protrusion arrays with a thickness of about 50 nm.

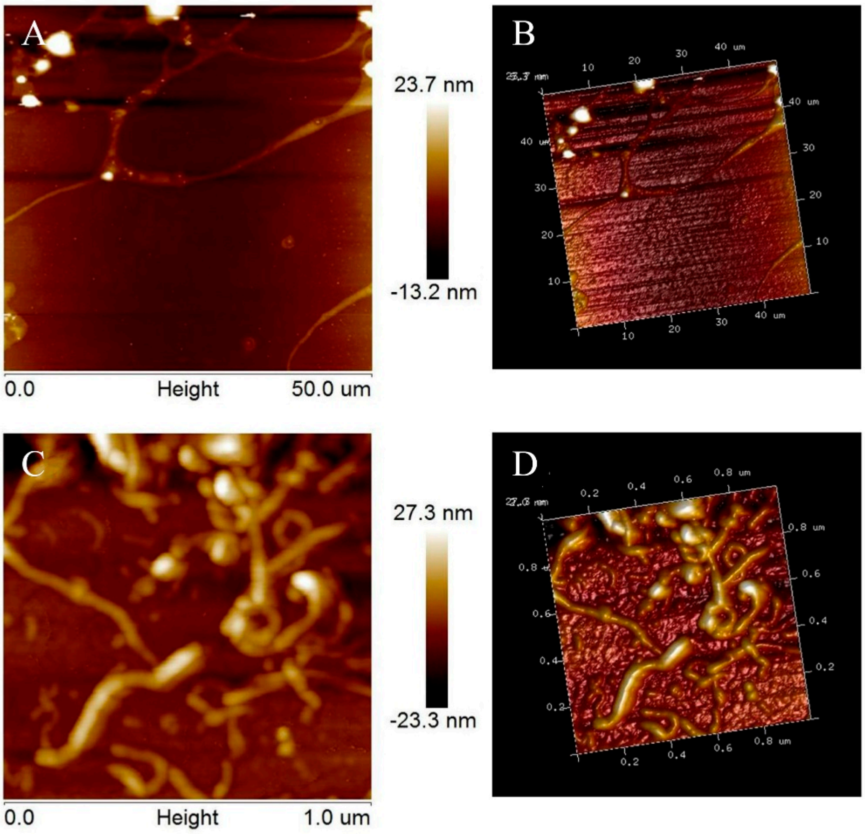

Figure S2. AFM images of commercial MWCNTs and acid-treated MWCNTs: 2D (A) and 3D (B) AFM images of raw MWCNTs; 2D (C) and 3D (D) AFM images of MWCNTs treated with mixed acids.

1.3. IR spectra of carboxylic MWCNTs

IR was used to analyze the types of functional groups at the open end of MWCNTs. Commercial MWCNTs do not show a characteristic peak in the range of 500-4000cm^{-1} (**Figure S3**, curve a). However, there is a characteristic peak at 1710cm^{-1} on curve b (acid-treated MWCNTs) that corresponds to the stretching vibration band of carbonyl in the carboxyl group (**Figure S3**, curve b). The result confirmed the existence of the carboxyl group in the acid-treated MWCNTs.

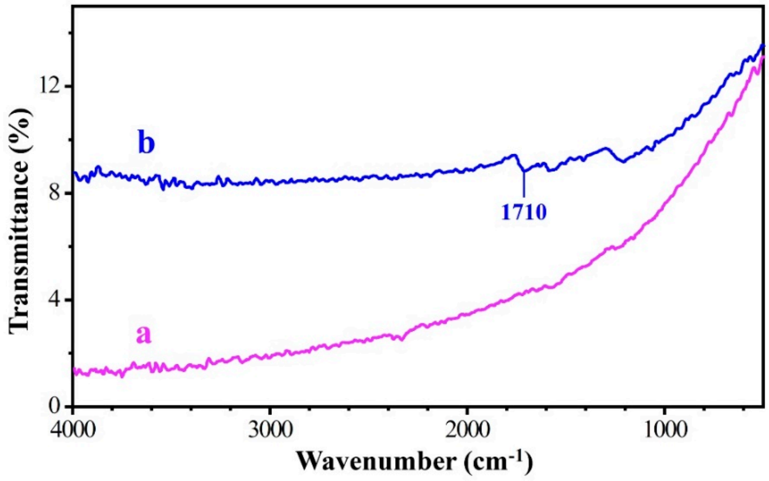

Figure S3. IR spectra of commercial MWCNTs (a) and acid-treated MWCNTs (b).

2. Optimization of experimental conditions

2.1. MWCNTs modification

The spacing of MWCNTs on the gold electrode surface depends on the reaction time of modification. Optimization reaction time was investigated by CC using RuHex as an electrochemical hybridization indicator. A quantum of 10 mg carboxylic MWCNTs and 20 mg coupling agent DCC were added to 10 mL of N, N- dimethylformamide (DMF) to ultrasonic disperse at room temperature for 10 min, and were then reacted at 60°C for a certain time. **Figure S4** shows the electric quantity increments (ΔQ) vs reaction time in the range from 3 to 12 h. The maximum ΔQ appeared at 9 h and the concentration of MWCNTs on the gold electrode surface increased with the passage of time for 9 h; after that, it decreased due to the rupture of the amide bond.

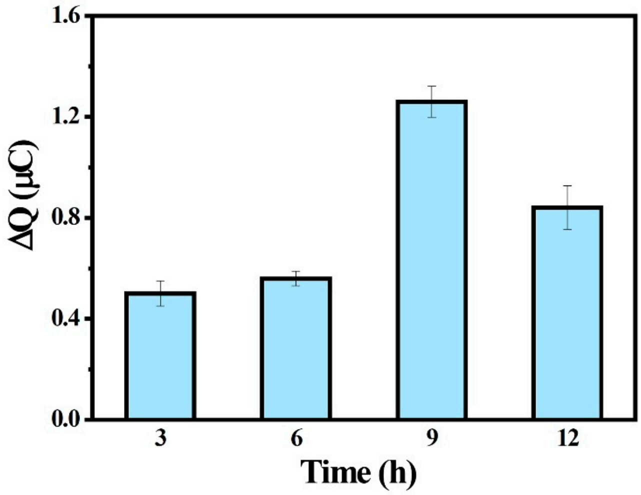

Figure S4. The effects of reaction time on the modification of MWCNTs.

2.2. Probe ssDNA immobilization

The loading of probe ssDNA was connected to the reaction time and the probe concentration. The ssDNA probe density on the surface of the modified electrode could be regulated by controlling the above two conditions.

MWCNTs/Cys/AuE were immersed in a mix solution of 1-ethyl-3- (3-dimethyl aminopropyl) carbodiimide (EDC) and N-Hydroxysuccinimide (NHS) at room temperature

for 30 min to activate carboxy located at the end of the tubes of the MWCNTs, and then cleaned with doubly distilled water and dried with nitrogen. After adding dropwise 4 μL 5 μM amino-modified probe ssDNA (P1) to the electrode's surface, the reaction time was set at 0.5-3.0 h with a 0.5 hour interval. The optimal reaction time is 1.5 h (**Figure S5A**). Under the above conditions, MWCNTs/Cys/AuE reacted with the probe ssDNA at different concentrations for 1.5 h; the maximum charge increments appeared at 5 μM (**Figure S5B**).

Generally, the increases in reactant concentration and reaction time would increase the density of the probe ssDNA on the surface of the modified electrode. Nevertheless, as shown in **Figure S5**, the charge increment ΔQ increased at first and then decreased as the reaction time and probe concentration increased. These experimental results showed that the density of the probe ssDNA fixed on the electrode surface is not as high as possible. If the density of the probe DNA is too high, the process of DNA hybridization would be affected due to the steric hindrance, and even heterogeneity between probes would be created, leading to low hybridization efficiency and reproducibility.

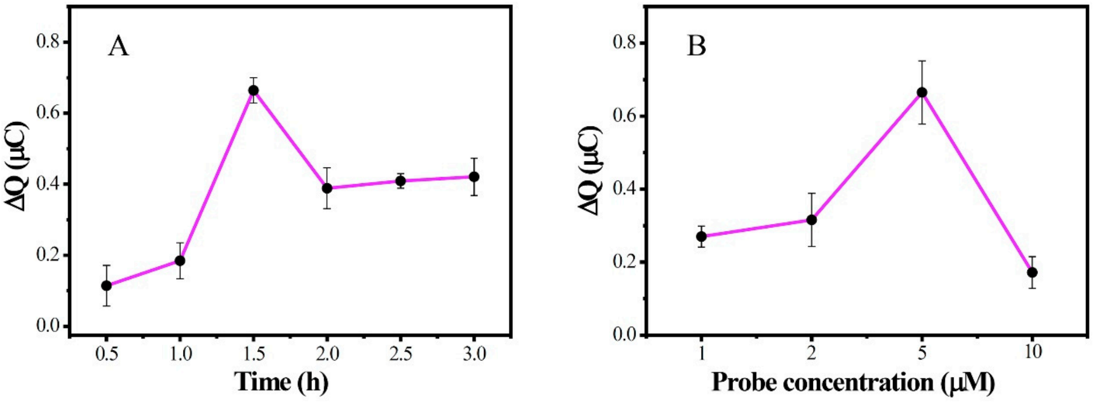

Figure S5. Effects of the reaction time (A) and probe concentration (B) of the probe ssDNA immobilization on the response of the biosensor.

2.3 DNA hybridization

As shown in **Figure S6**, the hybridization procedure was performed by immersing ssDNA/MWCNTs/AuE in 10 mM PBS containing 1nM complementary ssDNA for 1 h at different reaction temperatures in the range of 31°C to 46°C; the CC data show that 40°C was the optimal temperature. Setting the reaction temperature at 40°C (**Figure S5A**), and the reaction time in the range of 0.5-3 h, the result shows that the optimal reaction time is 1 h (**Figure S5B**).

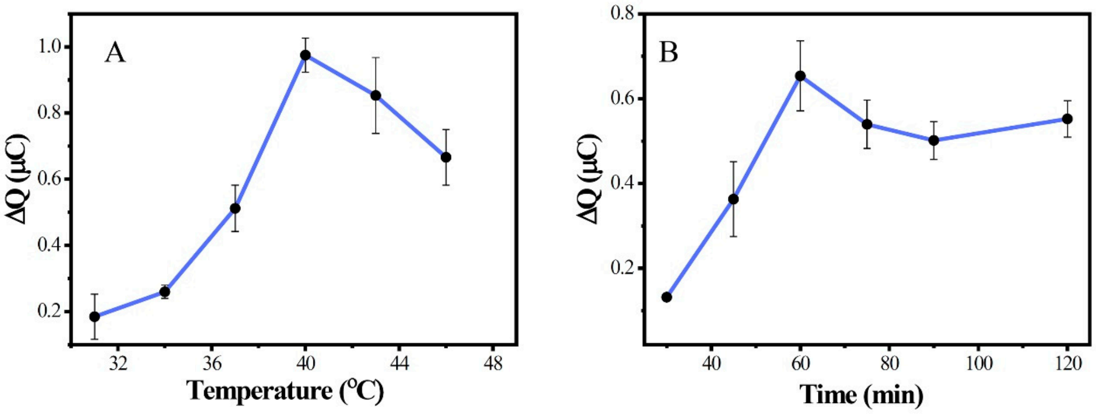

Figure S6. Effects of hybridization temperature (A) and hybridization time (B) on the response of the biosensor.
